# Self-Reported Intake and Circulating EPA and DHA Concentrations in US Pregnant Women

**DOI:** 10.3390/nu15071753

**Published:** 2023-04-04

**Authors:** Keri Lanier, Breanna Wisseman, Cody Strom, Carol A. Johnston, Christy Isler, James DeVente, Edward Newton, Roman Pawlak, Brittany R. Allman-Tucker, Samantha McDonald, Linda E. May

**Affiliations:** 1 School of Osteopathic Medicine, Campbell University, Lillington, NC 27546, USA; 2 Department of Kinesiology, East Carolina University (ECU), Greenville, NC 27858, USA; 3 Department of Kinesiology and Sport, University of Southern Indiana, Evansville, IN 47712, USA; 4 Department of Human Development and Family Science, East Carolina University (ECU), Greenville, NC 27858, USA; 5 Department of Obstetrics and Gynecology, East Carolina University (ECU), Greenville, NC 27858, USA; 6 Department of Nutrition, East Carolina University (ECU), Greenville, NC 27858, USA; 7 Department of Sport and Exercise Science, University of Central Arkansas, Conway, AR 72035, USA; 8 Kinesiology and Recreation, Illinois State University, Normal, IL 61790, USA; 9 East Carolina Diabetes and Obesity Institute, East Carolina University (ECU), Greenville, NC 27858, USA

**Keywords:** dietary assessment, food intake, polyunsaturated fatty acid, DHA, EPA, pregnancy

## Abstract

In the United States, pregnant women have low concentrations of docosahexaenoic acid (DHA) and eicosapentaenoic acid (EPA), which are essential for fetal development. Although maternal blood provides accurate polyunsaturated fatty acid (PUFA) concentrations, venipuncture is expensive and not always accessible. PUFA-containing foods consumption, both omega-3 ad omega-6 is supposed to reflect in the status (plasma, RBC, adipose tissue) of docosahexaenoic acid (DHA) and eicosapentaenoic acid (EPA). De novo synthesis of DHA and EPA during pregnancy is supposed to be higher compared to pre and/or post-pregnancy periods. Thus, this study aimed to determine the association between maternal self-reported dietary intake of foods high in DHA and EPA, along with vegetable oils as a source of omega-6 fatty acids, with maternal blood DHA and EPA concentrations. Pregnant women (13–16 weeks gestation) were recruited and asked to complete a food-frequency questionnaire (FFQ) and blood draw at enrollment and 36 weeks. Circulating concentrations of DHA and EPA were quantified and change scores were calculated. Correlations were done to determine associations between FFQ results and EPA/DHA maternal blood concentrations. Regression analyses were run to examine significant predictors of the main outcomes. Overall, PUFA-food consumption and RBC’s DHA levels decreased from early to late pregnancy; self-reported PUFA-rich food consumption positively correlated with DHA and EPA levels. DHA concentration was predicted by self-reported PUFA-rich oils (sunflower/soy/corn/olive) consumption, but EPA concentration was predicted by maternal BMI. These findings suggest that EPA and DHA consumption decreased across pregnancy and the FFQ can be utilized as an effective method for estimating PUFA blood concentration during pregnancy.

## 1. Introduction

In the United States (US), pregnant women usually have low ratios of omega-3 fatty acids to omega-6 fatty acids, due to a Western diet that prioritizes red meats, chicken, and corn oil, which exceeds the suggested omega-3s to omega-6s ratio of 1:4 up to 1:15 [1,2,3]. A diet high in cold-water fish, algae, and low intake of omega-6 fatty acids can help maintain the minimum suggested ratio, i.e., 1:4, of polyunsaturated fatty acids (PUFAs). This type of diet, high in omega-3 fatty acids, such as docosahexaenoic acid (DHA) and eicosapentaenoic acid (EPA), is important for the nervous system and health [4,5]. DHA and EPA play a critical role in fetal development, especially the fetal nervous system [4]. These PUFAs influence fetal brain development as well as inflammatory properties throughout the body [4]. DHA, in particular, is important for developing neuronal connections, neurogenesis, and protection from oxidative stress in utero [4]. For this reason, it is important to be able to accurately measure whole-body PUFA levels in pregnant women.

Previously, food-frequency questionnaires (FFQs) have been validated in Chinese men and women as well as in Australian women in late pregnancy [6,7]. Similarly, in Japan, there has been some utility established for assessing self-reported DHA and EPA via questionnaire, in early and late pregnancy [8]. However, these studies utilized only red blood cells (RBCs) in their analyses, which can only provide an assessment of long-term PUFA consumption habits [9,10]; whereas plasma levels provide a short-term assessment of PUFA levels and may more closely mimic PUFA food intake. However, this assessment has not been done previously in US women. Furthermore, studies in other countries varied in timepoints of assessments during pregnancy; and/or utilized a FFQ that was not inclusive of PUFA-containing foods. Foods such as fish, sunflower/soy/corn/olive oils, and almond/cashew milk contribute to PUFA consumption [11,12]. DHA and EPA are important fatty acids that play an integral role in fetal neurological development; therefore, it is imperative that healthcare providers are aware of maternal PUFA intake. While venipuncture sampling is a practical method for assessing maternal DHA and EPA blood concentrations, this process is invasive, costly, and time-consuming. With fetal brain development beginning in early pregnancy, it is important to have a rapid method for estimating maternal PUFAs, allowing for early intervention if levels are too low [13]. FFQs are non-invasive, easy to distribute and understand, and provide a rapid assessment of maternal food intake, thus, making them a low-cost, clinic- and patient-friendly alternative to venipuncture blood sampling. While previous research has been done in other populations, there is no literature validating a FFQ with DHA and EPA levels in the United States during early and late pregnancy. Therefore, the purpose of the present study was twofold: (1) to measure DHA and EPA levels in RBC and plasma in early and late pregnancy, and (2) to determine the association, and possible predictors, between self-reported consumption of PUFA-containing foods with DHA and EPA concentrations in maternal RBC and plasma in early and late pregnancy. We hypothesize that: (1) PUFA-rich foods, DHA, and EPA levels in plasma and RBCs will be similar in early and late pregnancy, and (2) there will be a positive correlation, and possible predictors, between self-reported PUFA-rich food consumption and circulating PUFA plasma, but not necessarily RBC, concentrations.

## 2. Methods

### 2.1. Study Design and Participants

The present study was a post hoc secondary analysis of a larger prospective randomized controlled trial designed to examine the influence of maternal exercise during pregnancy on fetal and infant health outcomes [14]. Participants were enrolled if they were 18–40 years old, able to communicate in English, ≤16 weeks pregnant, had a pre-pregnancy BMI of 18.5–39.9 kg/m^2^, and had a singleton pregnancy. All women were required to receive written clearance from an obstetric provider to participate in the study. Participants were excluded from the study if they had pre-existing diabetes mellitus, hypertension, cardiovascular disease, co-morbidities known to affect fetal growth and well-being (e.g., systemic lupus erythematosus), or used tobacco, alcohol, and illicit drugs. All protocols were approved by the East Carolina University Institutional Review Board. Clinical Trial Registry is #NCT03517293. Written informed consent was obtained from each participant.

Pre-screening eligibility questionnaires and neonatal electronic health records were used to determine maternal age, gravida, parity, pre-pregnancy weight and height, education level, gestational weight gain (GWG), and gestational age (weeks). Height was measured using a stadiometer and weight was collected using a standard scale at 16 and 36 weeks gestation. Pregnancy weight was assessed at the same time points using a calibrated digital scale. Pre-pregnancy weight was self-reported at enrollment (≤16 weeks). A standardized equation was used to calculate BMI at each time point [15]: $\mathrm{BMI}=\left((\mathrm{weight}\;\left(\mathrm{kg}\right)\right)÷\left(\left[\mathrm{height}\;\left(m^{2}\right)\right]\right))$; BMI classification used standard cutoffs: normal weight: 18.5–24.9 kg/m^2^; overweight 25–29.99 kg/m^2^; obese ≥ 30 kg/m^2^.

### 2.2. Maternal Food-Frequency Questionnaire

Participants (N = 47) were asked to complete a food-frequency questionnaire (FFQ) at enrollment (13–16 weeks) and 36 weeks gestation to obtain self-reported PUFA levels. The FFQ asked women to specifically report foods, such as PUFA-rich foods, consumed during pregnancy [16]. The women were asked to report the frequency of consumption of foods based on the scale: 1—rarely or never eat the food, 2—eat the food once every 2 weeks, 3—eat the food 1–3 times/week, 4—eat the food 4–7 times/week, or 5—eat the food more than once per day. The individual PUFA-rich foods (white-flesh fish, other fish (e.g., salmon), almonds) were rated on the 5-point Likert scale [11,12]. Both the polyunsaturated margarines and sunflower/soy/corn/olive oils were rated on “Yes, you consume” or “No”; these two dichotomous measures were converted to No = 0 and Yes = 1 for analysis. All individual PUFA-rich food column numerical values were then summed for a PUFA summary score for each participant during pregnancy.

### 2.3. Maternal Plasma and RBC Collection and Analysis

A fasting venous blood sample was collected from women at enrollment (13–16 weeks) and 36 weeks gestation. All samples were completed following a ≥8 h fast and collected between 6–9 a.m. Blood was centrifuged and stored using standard procedures as described previously [14]. Both blood plasma and RBCs were utilized for analysis as plasma provides a representation of recent concentrations and RBCs provide longer term (~120 days) concentrations [9,10].

### 2.4. Maternal DHA and EPA

Chemicals and reagents: Optima grade acetonitrile, water, formic acid, methanol, and isopropanol were purchased from Fisher Scientific (Hampton, NH, USA).

Preparation of calibration and quality control standards: Working stock solutions were prepared for calibrators. Samples were screened for quality control (QC). Calibration curves were generated from 0.01–7.5 mg/mL. A positive cutoff limit was established at 10 mg/mL. Low and high QC samples were prepared by the addition of 10 or 500 ng/mL and were fortified as a QC solution.

Targeted LC/MS: An Agilent Poroshell (Agilent Technologies, Santa Clara, CA, USA) 120 EC-C8, 3 × 100 mm 2.7 µm column was used for separation of the analytes on an Exion HPLC. The column temperature was maintained at 32 °C. A gradient was used to separate the compounds using mobile phase A: 95:5 water with 0.1% formic acid:acetonitrile and mobile phase B: acetonitrile. A linear gradient was performed as follows: 0% B for 2 min, 90% B for 9 min, 90% B for 1 min, 0% B for 1 min, hold at 0% B for 5 min for a total run time of 18 min. The flow rate was 0.3 mL/min and 5 µL of sample was injected. MS-MS analysis was conducted using an AB SCIEX 3200 (Danaher Corporation, Toronto, ON, Canada) triple quadrupole mass spectrometer. The mass spectrometer was in negative ionization mode and analysis was conducted using multiple reaction monitoring (MRM). The source parameters were set to a curtain gas 50 psi, heater gas 50 psi, ion spray voltage 5500 V, and source temperature 500 °C. The instrument parameters were optimized using direct infusion of each analyte using a split tee injection with the LC flow. SCIEX Analyst software (v.1.6.2—Sciex Applied Biosystems, Framingham, MA, USA) was used for instrument control. Confirmation analysis was performed using MultiQuant where the calibrators and quality controls were carried through the same processes as the specimens being tested. Least squared regression with 1/x weighing was used to evaluate the linearity with adequate compensation for heteroscedasticity during all experiments.

#### 2.4.1. Solid Phase Extraction (SPE)

DHA and EPA were extracted from RBCs [17,18]. Plasma samples were prepared following a similar method. Plasma samples were prepared utilizing a 3.9:1 Optima grade H_2_O (Fisher Scientific, Hampton, NH, USA) to plasma solution, were vortexed, and homogenized. Aliquots of 490 mL of plasma solution were diluted to a 1 mL solution with 500 mL of methanol (MeOH) and 10 mL deuterated DHA (DHA-d5) and EPA (EPA-d5) internal standard solution. Immediately following solution preparation, both the RBC and plasma solutions were centrifuged at 13.2 rpm for 20 min. Strata-X reversed-phase SPE columns (Phenomenex, Torrance, CA, USA) and positive pressures (1 to 25 psi) were used to extract the supernatants on a Biotage Pressure+ manifold (Biotage, Charlotte, NC, USA). Columns were conditioned with 1 mL of MeOH and equilibrated with 2 mL of H_2_O. Supernatants were loaded onto the conditioned columns and were washed with 1 mL of 10:90 MeOH:H_2_O. The organic fraction of metabolites was collected by loading 1 mL of MeOH and 1 mL of 60:20:20 Acetonitrile(ACN):MeOH:IPA in duplicate, then evaporated using a steady flow of nitrogen gas and heat of 40 °C. Samples were reconstituted in 100 mL of 50:50:0.01 H_2_O:MeOH:formic acid and the solution was transferred into 100 μL autosampler vials for analysis on an AB SCIEX 3200 triple quadrupole mass spectrometer. A processed blank was extracted using the same method. All samples were stored and run in batches.

#### 2.4.2. Calibration Curve

A calibration curve was used to quantify the analytes. Stock solutions were prepared in ethanol with DHA and EPA standards, each at a concentration of 25 mg/mL. DHA and EPA standard solutions were prepared by serial dilution of the stock solutions in ACN to create primary standards at 0.01, 0.05, 0.1, 0.5, 1, 2.5, 5.0, and 7.5 mg/mL. Deuterated DHA and EPA were used as internal standards prepared at 0.5 mg/mL in ethanol. The deuterated DHA and EPA solution controlled for extraction recovery, injection of the mass spectrometer, and ionization variability. The stock solutions were processed and extracted using the same method of the plasma solution.

#### 2.4.3. Liquid Chromatography/Mass Spectrometry (LC/MS/MS)

Extracted samples were run on an AB SCIEX 3200 triple quadrupole mass spectrometer in negative ionization mode using previously published methods [17,18,19]. Change values for DHA and EPA were calculated between timepoints by subtracting 16-week concentrations from 36-week concentrations. 

### 2.5. Statistical Analyses

Summary statistics were run for maternal descriptors, PUFA-rich foods from the FFQ, as well as levels from maternal blood. For FFQ, data was converted into the average number of times consumed per week as such: rarely or never eat the food = 0, eat the food once every 2 weeks = 0.5 times per week, eat the food 1–3 times/week = 2 times per week, eat the food 4–7 times/week = 5.5 times per week, or eat the food more than once per day = 7 times per week. Both the polyunsaturated margarines and sunflower/soy/corn/olive oils were rated on for whether they were used with foods (i.e., breads, vegetables) and with cooking. For both margarine and oil responses: Yes = 7 per week, No consumption = 0 per week; thus, the potential score for margarine and oils ranged from 0 to 14 considering use with food and with cooking to both questions. The summation of these columns provided the PUFA summary score for each participant during pregnancy. Data are reported as mean ± standard deviation (SD) unless data was not non-normally distributed then median (minimum, maximum) were reported. Difference values were determined by subtracting the 16-week value from the 36-week value for maternal lipid levels. Based on difference values (16 to 36-week change scores), all participants were coded as improved (increased score), no change, or decreased score for all DHA, EPA, and FFQ summary data. Participants that had a decreased DHA or EPA blood value and decreased FFQ value were coded as non-responders, while those with increased DHA or EPA blood values with increased FFQ values were coded as responders. Thus t-tests were completed to compare non-responders with responders. Spearman’s rank correlation tests were performed to determine relationships between maternal self-reported consumption of PUFA-rich foods with measured values from blood. Linear regressions were done to determine if self-reported values were predictors of maternal blood levels. Significance level was set a priori at 0.05 and SPSS was used for all analyses (SPSS 25.0 Chicago, IL, USA).

## 3. Results

Study Population. For this analysis, 156 pregnant women expressed interest; of these women, 145 were qualified and consented. Throughout the study, 38 participants were lost-to-follow-up with participant refusal (*n* = 6), moved, no time or lost interest (*n* = 29), discontinued due to drug use (*n* = 1), discontinued due to bed rest (*n* = 1), or miscarried (*n* = 1). Of the remaining 107 participants, 60 were excluded due to missing data for plasma, RBCs, and/or incomplete questionnaire data. Thus, a final sample of 47 pregnant women completed 16-week and 36-week FFQs and venipunctures for this post hoc analysis. On average, participants were 31 years old, had a mean BMI in a healthy range, with appropriate GWG, and delivered full-term healthy babies free from congenital issues (Table 1). The median response from the FFQ was that most women did not consume white fish, other fish, almond/cashew milk, or use polyunsaturated margarine on a regular weekly basis at 16 and 36 weeks gestation; at 16 and 36 weeks, participants reported using oil on foods and for cooking (Table 2). Overall, the PUFA summary decreased from 16 to 36 weeks gestation (Table 2).

### 3.1. EPA and DHA Status

Both maternal RBC DHA concentration and plasma DHA concentration decreased from 16 to 36 weeks. In contrast, maternal RBC EPA concentration increased from 16 to 36 weeks (Table 3). When comparing participants with overall decreased DHA or EPA blood values and decreased FFQ values (non-responders) to those participants with overall increased DHA or EPA blood values and increased FFQ values (responders), participants that have overall decreased DHA and EPA blood values have significantly increased GWG (*p* = 0.02) with no differences in maternal age, gravida, and pre-pregnancy BMI.

### 3.2. Correlation Analysis

There were no correlations between maternal DHA or EPA in blood compared to self-reported fish consumption. There were moderate positive correlations between self-reported almond/cashew milk consumption at 36 weeks gestation with 36-week plasma concentration of DHA (*p* = 0.01, r = 0.582) as well as with the change value of plasma DHA (*p* = 0.041, r = 0.473) from 16 to 36 weeks (Table 4). Similarly, self-reported 36-week DHA- and EPA-rich oil consumption (sunflower/soy/corn/olive) moderately correlates with 16-week EPA on RBCs (*p* = 0.04, r = −0.306), 36-week plasma DHA (*p* = 0.046, r = 0.464), and the change in plasma DHA (*p* = 0.02, r = 0.519) from 16 to 36 weeks (Table 4). Lastly, self-reported 36-week PUFA-rich summary score moderately correlates with 36-week plasma DHA (*p* = 0.01, r = 0.566), and the change in plasma DHA (*p* = 0.01, r = 0.567) from 16 to 36 weeks gestation (Table 4).

### 3.3. Regression Analysis

We found predictive models for plasma and RBC concentrations of EPA and DHA (Table 5). Controlling for gravida and 16-week self-reported PUFA-rich food summary, 16-week maternal BMI (*p* = 0.01) predicted 16-week maternal EPA on RBCs (Table 5); this suggests that changes in 1 unit of BMI correlates with about 2 ng/dL of maternal EPA, which may not be clinically meaningful. When controlling for gravida and GWG, 36-week self-reported PUFA oil consumption (*p* = 0.02) significantly predicted 36-week plasma DHA (Table 5); this suggests that if women added 2–3 servings/day of PUFA oil, this could increase plasma DHA by 10 ng/dL, which could result in clinically meaningful differences. Other models that approached significance or did not have any significant individual predictors are not shown.

## 4. Discussion

The purpose of the study was: (1) to measure DHA and EPA levels from maternal blood in early and late pregnancy, and (2) to determine the association, and possible predictors, between self-reported consumption of PUFA-containing foods with DHA and EPA concentrations in maternal blood in early and late pregnancy. We hypothesized that: (1) PUFA-rich foods, DHA, and EPA levels will be similar in early and late pregnancy, and (2) there will be a positive correlation, and possible predictors, between self-reported PUFA-rich food consumption and circulating PUFA concentrations which was consistent with our findings. Our main findings were as follows: (1) DHA levels in maternal blood, and self-reported PUFA-food average weekly consumption, decreased from 16 to 36 weeks gestation; (2) self-reported PUFA-rich food average weekly consumption positively correlates with measured DHA and EPA levels in blood; and (3) DHA, but not EPA, concentration in blood was predicted by self-reported PUFA oils consumption.

We found both self-reported PUFA-food weekly consumption and measured DHA levels decreased from early to late pregnancy, which was different than expected. This suggests that there is decreased maternal consumption of PUFA-rich oils as pregnancy progresses. Previous literature on this topic reports conflicting results. One study notes that serum fatty acid concentrations of DHA, EPA, and total omega-3 PUFA’s increase from the first to second trimester, with a slight, but continued, increase from the second to third trimester, which was attributed to an increase in lipids transported across the placenta after 20 weeks gestation [20]; however, our study was of US women with MS technology and the previous findings were in Brazilian women and LC technology, possibly explaining the difference in findings. Similar to our findings, another study reported decreasing levels of maternal plasma levels of DHA from 27 weeks gestation until delivery [21]. One further longitudinal study from Spain found an increase of total omega-6 PUFA’s, a decrease of EPA, and no significant change of DHA, from the first to the third trimester of maternal plasma [22]. In the present study, decreasing concentrations of DHA on RBCs and in plasma were found from early and late pregnancy. The decreasing levels of DHA may be explained by a decrease in consumption of DHA and EPA-containing oils throughout the pregnancy. Previous studies have focused on the intake of fish throughout pregnancy [8]; whereas the present study suggests a potential recommendation of increasing maternal PUFA-rich oil intake during pregnancy. Since those with generally decreased DHA or EPA in blood and on the questionnaire have significantly increased GWG, further research is needed to determine why these women may have an overall decrement in nutrition quality as pregnancy progresses.

As we hypothesized, we found self-reported 36-week PUFA-rich average weekly food consumption positively correlates with DHA and EPA levels at 36 weeks. Similar to the present study, previous research by Kobayashi et al. found a correlation between food intake and serum levels of EPA (R = 0.37) and DHA (R = 0.27) during pregnancy [8]. This study utilized a FFQ which had users rate foods such as fish, shellfish, and other fish products based on consumption and portion size during early (8–14 weeks) and late (26–35 weeks) gestation in Japan [8]. This differed from the current study in which correlations were analyzed for specific foods, allowing further assessment of which food items correlate best with the blood sample findings. Furthermore, the FFQ utilized in the Kobayashi et al. study has limited accuracy in measuring cooking oil as a possible source of PUFA intake; this is a limitation that our study was able to address [8]. Other research evaluated the validation of a FFQ measuring PUFA status in non-pregnant adults [7]. This study found a positive correlation between self-reported dietary DHA intake, specifically fish, with plasma DHA, but no correlation between plasma EPA [7]. The discrepancy between these findings and our findings of a positive correlation with both plasma DHA and EPA could be due to the type of FFQ used. The FFQ utilized in the previously mentioned research emphasized PUFA status from fish consumption, whereas the questionnaire utilized in the present study emphasized PUFA-rich foods, such as fish, oils, and margarine. These details provide validation regarding the participant’s diet relative to a sensitive measure of EPA and DHA intake, thus accounting for the difference in results.

Finally, we found that 36-week DHA concentration was predicted by 36-week self-reported PUFA oil consumption, but EPA concentrations were predicted by maternal BMI. These findings suggest that maternal DHA levels could potentially be estimated by FFQ self-reported PUFA oil levels during pregnancy. This would provide a non-invasive method of assessing late pregnancy PUFA status, to ensure recommended levels are met, as an essential part of proper fetal development. Interestingly, maternal EPA concentrations were predicted with a negative association with maternal BMI. Similar to our findings, a study by Young et al. reported a negative association between BMI of non-pregnant women and omega-3 index [22]. The negative association was proposed to be due to altered metabolic pathways in the absorption and utilization of omega-3 fatty acids in women with obesity compared to healthy BMI [22]. The similar findings between our study and that of Young et al. suggests that the utilization of EPA is similar in non-gravid and gravid women. Further research is needed to accurately define the role of maternal BMI, most likely adiposity status, in pregnant women and EPA concentrations.

The strengths of our study include the unique comparison of early and late pregnant women with blood samples and questionnaire data. While our study provides valuable insights, this research is not without its limitations. First, the small sample and non-normally distributed variables, may influence linear regression analysis; however, the uniqueness of the data argue for further investigation with a larger sample to enable the accuracy and efficiency of linear regression estimates. With more data, the type 2 error in the study would be reduced, leading to a higher sensitivity and greater generalizability of the outcomes. Furthermore, as with any self-report method for assessment, there is a potential for self-reported bias when responding. Women may feel compelled to alter their answers based on what they think they should be consuming, not what they consumed. It is important to note that some of the foods contained in the FFQ contained omega-3 and omega-6 fats; therefore, further research needs to explore how women’s self-reported response relates to blood levels of both types of unsaturated fats. However, given the correlation between the self-reported data and the direct measurement of blood variables and the low-cost, patient- and clinic-friendly use of FFQ relative to the use of venipuncture, this warrants further investigation.

Future research should expand upon the current study by assessing neonatal outcomes according to self-reported PUFA status. Furthermore, more research can be done to optimize the use of the FFQ and address a larger, more diverse pregnant population. The FFQ from the present study can be implemented in OB/GYN clinics with the goal of providing patient knowledge on PUFA-containing foods and creating obtainable goals for patients on the amount of those foods to consume. Further research should focus on overall nutrition quality in those women who have trends of increased GWG. This may create more education for patients and create better outcomes for neonates.

## 5. Conclusions

In conclusion, the present study found that average weekly PUFA consumption and blood levels seem to decrease throughout pregnancy. Importantly, the Sheffield FFQ seems to be an effective method for estimating late pregnancy DHA and EPA blood levels. This research allows for a compelling and non-invasive method for estimating DHA and EPA concentration in pregnant women, especially the third trimester. Furthermore, by utilizing a FFQ, women can be aware of their DHA and EPA status, thus, allowing a simpler approach for patients and clinical professionals to track PUFA intake throughout pregnancy. The present study provides insight into an easy, cost-effective method for estimating DHA and EPA status in pregnant women but warrants further nutrition analysis. Overall, the Sheffield FFQ might provide a noninvasive, low-cost method to estimate DHA and EPA status, especially in late pregnancy; however, further investigation with a larger sample is warranted.

## Figures and Tables

**Table 1:** Maternal descriptors.

	Mean ± SD
Age (years)	30.56 ± 2.63
Gravida ^a^	2 (1, 4)
Parity ^a^	0 (0, 2)
Pre-pregnancy BMI (kg/m^2^) ^a^	23.47 (20.5, 42.5)
16-week BMI (kg/m^2^) ^a^	24.4 (21.5, 43.9)
36-week BMI (kg/m^2^) ^a^	28.1 (24.76, 43.3)
Education (years) ^a^	19 (13, 23)
Gestational weight gain (kg)	12.35 ± 6.42
Gestational age (weeks)	39.83 ± 1.05

All values reported as mean ± SD. ^a^ Values reported as median (minimum, maximum) and used a Mann–Whitney U test due to non-normal distribution. BMI: body mass index.

**Table 2:** Self-reported frequency of food consumption per week at 16 and 36 weeks gestation.

Food Intake Categories	16 Weeks	36 Weeks
White fish (servings/wk)	0 (0, 2)	0 (0, 0.5)
Other fish (servings/wk)	0 (0, 0.5)	0 (0, 0.5)
Milk (almond or cashew) (servings/wk)	0 (0, 8)	0 (0, 8)
Polyunsaturated margarine (servings/wk)	0 (0, 0)	0 (0, 0)
Oil number (servings/wk)	7 (0, 14)	7 (0, 14)
PUFA summary ^a^ (servings/wk)	9.25 ± 7.4	7.8 ± 7.11

All values reported as median (minimum, maximum) and used a Mann–Whitney U test due to non-normal distribution. ^a^ Values reported as mean ± SD. PUFA: polyunsaturated fatty acid.

**Table 3:** Maternal blood EPA and DHA concentrations at 16 and 36 weeks gestation and the difference from early to late pregnancy.

	16 Weeks	36 Weeks	Difference
RBC DHA (ng/dL)	1279.56 ± 631.08	1187.0 (450.2, 6012.0) ^a^	−42.2 (−2006.0, 5501.8) ^a^
Plasma DHA (ng/dL)	448.02 ± 186.03	415.7 ± 193.89	−32.32 ± 276.17
RBC EPA (ng/dL)	715.18 ± 426.76	930.8 (165.1, 4656.0) ^a^	10.95 (−395, 3867) ^a^

All values reported as mean ± SD. ^a^ Values reported as median (minimum, maximum) and used a Mann–Whitney U test due to non-normal distribution. RBC: Red Blood Cell, DHA: docosahexaenoic acid, EPA: eicosapentaenoic acid.

**Table 4:** Correlation between self-reported PUFA-rich food consumption and maternal blood and plasma DHA or EPA concentrations.

Measure	*p*-Value	Pearson Correlation
16-week PUFA-rich foods summary		
RBC EPA difference	0.055	0.282
36-week milk (almond or cashew) consumption		
36-week plasma DHA	0.009	0.582
Difference plasma DHA	0.041	0.473
36-week PUFA oil consumption		
36-week plasma DHA	0.046	0.464
Difference plasma DHA	0.023	0.519
16-week RBC EPA	0.037	−0.306
36-week PUFA-rich foods summary		
36-week plasma DHA	0.012	0.566
Difference plasma DHA	0.011	0.567

**Table 5:** Regression models to predict DHA and EPA levels in maternal blood.

	*p*-Value	95% CI Lower Bound	95% CI Upper Bound	Beta Value	STD Error
**16-week RBC EPA (Model 1)**	*p*-value = 0.045 * Adjusted R^2^ = 0.111				
16-week PUFA-rich food summary	0.65	−30.641	19.232	−0.069	12.365
16-week BMI	0.01 *	−53.157	−6.463	−0.368	11.577
**Difference in RBC EPA (Model 2)**	*p*-value = 0.046 * Adjusted R^2^ = 0.154				
Age	0.08	−150.318	9.005	−0.27	39.316
Education	0.08	−358.107	22.805	−0.321	93.997
16-week PUFA-rich food summary	0.23	−25.153	99.696	0.189	30.809
**36-week plasma DHA (Model 3)**	*p*-value= 0.044 * Adjusted R^2^ = 0.333				
Gestational weight gain	0.09	−0.878	11.674	0.393	2.926
36-week other fish consumption	0.051	−1.07	758.032	0.443	176.964
36-week PUFA oil consumption	0.02 *	2.956	35.063	0.548	7.485

BMI: body mass index; PUFA: polyunsaturated fatty acid. Bolded headers indicate significant models. * *p* < 0.05. Non-significant measures in each regression model includes: Model 1: gravida; Model 2: gravida, 16-week BMI; Model 3: gravida.

## Data Availability

De-identified data can be shared upon request to the corresponding author.
